# ‘Educate them early’—antimicrobial stewardship in schools using e-Bug: a brief review of online resources ‘KS2: Antibiotics’ and ‘KS3: antibiotic use and antimicrobial resistance’

**DOI:** 10.1093/jacamr/dlag041

**Published:** 2026-03-31

**Authors:** Helen Callaby, Lyra Joyohoy, Alishah Haider, Victoria Mycock, Rachel Mennie, Jill King, Fiona McDonald, Suzanne Brittain, Vhairi Bateman

**Affiliations:** The School of Medicine, Medical Sciences and Nutrition, University of Aberdeen, Aberdeen, UK; Aberdeen Royal Infirmary, Aberdeen, UK; The School of Medicine, Medical Sciences and Nutrition, University of Aberdeen, Aberdeen, UK; The School of Medicine, Medical Sciences and Nutrition, University of Aberdeen, Aberdeen, UK; The School of Medicine, Medical Sciences and Nutrition, University of Aberdeen, Aberdeen, UK; Infection Prevention and Control, Aberdeen Royal Infirmary, Aberdeen, UK; Medical Paediatrics, Royal Aberdeen Children's Hospital, Aberdeen, UK; Pharmacy, Aberdeen Royal Infirmary, Aberdeen, UK; Pharmacy, Aberdeen Royal Infirmary, Aberdeen, UK; Infectious Diseases and Medical Microbiology, Aberdeen Royal Infirmary, Aberdeen, UK

## Abstract

**Background and objectives:**

Antimicrobial resistance (AMR) is a growing threat to public health globally. The UK Health Security Agency (UKHSA) national action plan recommends a focus on public engagement and education about the risk of exposure to antimicrobials. The e-Bug programme, operated by UKHSA, provides freely available, evidence-based resources that empower teachers and pupils to learn about bacterial illnesses and safe antibiotic use. To review online education resources (e-Bug) for AMR as a resource for teaching primary-aged pupils about the topic. To establish a pilot programme of antimicrobial education for school children, taught by medical and postgraduate students from the University of Aberdeen, using the e-Bug online resource.

**Methods:**

A 1 h in-person training session was provided, led by members of the antibiotic management team (AMT), using e-Bug resources for lesson plans. Schools from within the local catchment area of the highest prescribing GP practices were targeted. Funding of £1000 was received from the University of Aberdeen Development Trust Student Fund.

**Results:**

Seventeen volunteers were trained, and 12 went on to complete visits. Five primary schools and one secondary school were visited. Twelve classes across the schools were taught, reaching approximately 300 pupils. Lesson plans are reviewed in detail in this article. Volunteers enjoyed the sessions and found them useful and rewarding. School teachers found the lessons informative and enjoyable for the pupils, and volunteers would be welcomed again.

**Conclusions:**

Using the e-Bug resource is relatively easy, straightforward and enjoyable for both teachers and pupils. Community outreach led by NHS antimicrobial teams is feasible, and incorporating university students to provide the teaching is a success.

## Introduction

Antimicrobial resistance (AMR) has been growing globally and is now one of the top global public health concerns.^1^ Raising and promoting public awareness plays an essential role in helping to tackle this around the world. The UK Health Security Agency (UKHSA) national action plan recommends a focus on public engagement and education about the risk of exposure to antimicrobials.^2^ The e-Bug programme, operated by the UKHSA, aims to address this growing concern by providing freely available, evidence-based resources that empower teachers and pupils to learn about bacterial illnesses and safe antibiotic use.^3^

e-Bug is an online, educational platform operated by the UKHSA. It is accredited by The Association for Science Education and includes lesson plans that have been developed in partnership with teachers and scientists. The aim is to educate children of different school ages about microbes, antimicrobial resistance and more.

## Methods

The antimicrobial management team (AMT) at Aberdeen Royal Infirmary recognized a gap between the recommendation of the UKHSA national action plan and the use of e-Bug. Therefore, they established a pilot programme of antimicrobial education for school children in Aberdeen, taught by medical and postgraduate students from the University of Aberdeen. Volunteer teachers were recruited using student e-newsletters and email. A 1 h in-person training session was provided, led by the AMT. e-Bug was used as the basis for a lesson plan, using the resources described below. Schools from within the local catchment area of the highest prescribing GP practices were targeted, and classes were taught of pupils aged 8–12. Funding of £1000 was received from the University of Aberdeen Development Trust Student Fund, to provide lesson resources, branded uniform and travel for volunteers.

The e-Bug’s key educational tools used and reviewed here are the ‘KS2: Antibiotics’, designed for Key Stage 2 pupils aged 7–11 years, and ‘KS3: Antibiotic Use and Antimicrobial Resistance’, designed for the education of Key Stage 3 pupils aged 11–14. The tools are UK based, which is a high income setting are UK based (high income).

## Results

**Figure 1.**
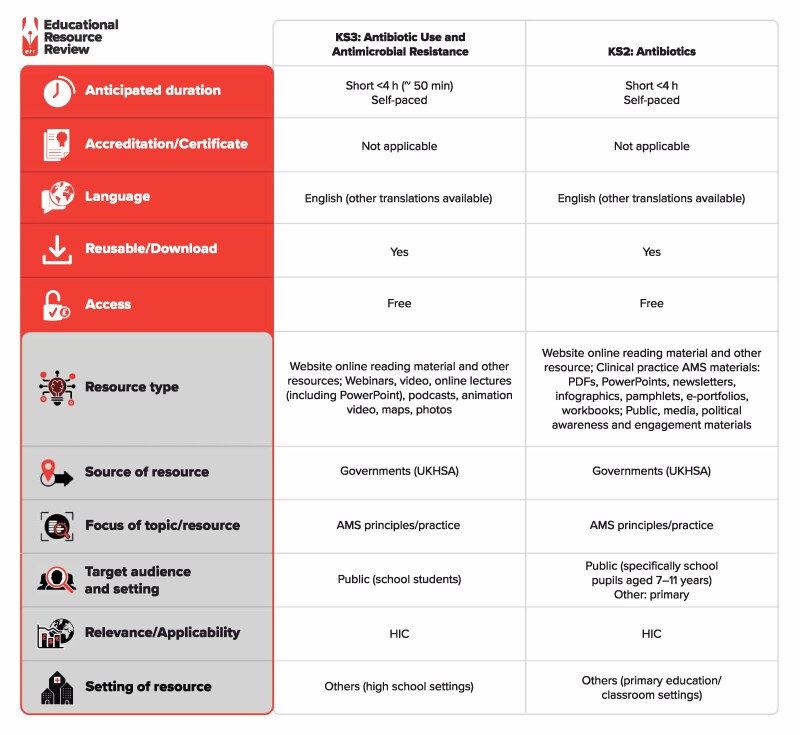
Summary of the resources reviewed. ‘KS2: Antibiotics’ (https://www.e-bug.eu/en-xi/ks2-antibiotics) and ‘KS3: Antibiotic Use and Antimicrobial Resistance’ (https://www.e-bug.eu/ks3-antimicrobial-resistance). The full classification scheme is available at http://bsac.org.uk/wp-content/uploads/2019/03/Educational-resource-review-classification-scheme.pdf. HIC, high-income countries.

Seventeen volunteers were trained and 12 went on to complete visits. Five primary schools and one secondary school were visited. Twelve classes across the schools were taught, reaching approximately 300 pupils. Each resource will be reviewed in turn here and summarized in Figure 1.

### KS2: Antibiotics

This resource contains a comprehensive set of materials, including a lesson plan, PowerPoint presentation, comic strips, teacher discussion points, antibiotics flashcards, and a word-mix-up game sheet. It is available in multiple downloadable formats, including pdf, Word and PowerPoint, allowing teachers to adapt the materials to suit different learning environments and abilities. The resource is free to access and requires no registration, ensuring it can be used widely in schools and other educational settings.

The lesson introduces the concept of antibiotics and supports teacher-led discussion and debate to help pupils understand that most common viral infections improve with simple measures such as rest and hydration. The main activity uses a comic strip that presents an engaging story to illustrate these ideas, prompting students to reflect on what they have learned through guided discussion. Extension activities, including flashcards and a word-mix game, provide further opportunities for pupils to consolidate their understanding through interactive learning.

The strengths of this lesson package lie in its accessibility, clarity and practicality. It is free, easy to navigate and can be downloaded in full for offline use. The structured lesson plan and adaptable teaching materials allow educators to modify the content according to class size, ability level and time available. The interactive nature of the comic strip and supporting games encourages active learning and reinforces understanding through participation. The design of the materials is visually engaging, and the key messages are reinforced throughout the lesson, ensuring that pupils can recall and apply what they have learned.

However, there are some limitations to consider. The lesson is designed for the UK Key Stage 2 curriculum, which may restrict its direct applicability in other educational contexts without modification. Although the materials are available in digital formats, there is no interactive online version or built-in quiz, which limits engagement for schools increasingly using virtual learning platforms.

Despite these limitations, the lesson remains a good example of how basic evidence-based educational materials can contribute to public health education and antimicrobial stewardship. Its free availability and engaging content make it a practical and valuable tool for teachers.

In summary, the ‘KS2: Antibiotics PowerPoint and Lesson Plan’ is a highly accessible, user-friendly and well-constructed educational resource that promotes early understanding of antibiotics and antimicrobial resistance.

### KS3: Antibiotic Use and Antimicrobial Resistance

The goal of this lesson is to begin introducing the concept of AMR with a structured and easily accessible lesson plan. Learning outcomes of the resource highlight the aim of demonstrating that antibiotics only treat bacterial infections, and how common illnesses such as the common cold don't require antibiotics.

The resource was implemented at a stage when the pupils had recently gained a foundational knowledge about microorganisms. A PowerPoint was provided, and supporting materials included teacher and student worksheets that could be downloaded. Activities were integrated as the class progressed to assess pupil understanding; the idea for this originated from the ‘Antibiotics Can/Can’t’ student worksheet provided by the module.

Although the worksheet prompted individual responses and input, questions were used instead for more interaction. Coloured cards were handed to each student to raise depending on the AMR question: Red for ‘NO’ and Green for ‘YES’. A debate activity recommended by the e-Bug PowerPoint; ‘I’m a Scientist’ was utilized to emphasize more collaborative learning, allowing students to engage in critical thinking and dialogue among the class.

## Discussion

The benefits of the e-Bug resource are its engaging and interactive format, which captures learners’ attention and promotes active participation. It is user-friendly, which allows users to locate the resources efficiently, and has structured lesson plans, following a logical learning progression with very clear instructions.

There are limitations in that although UK labelled, its modules are tailored for the English National Curriculum and it requires adjustments for other education systems such as Scotland’s Curriculum for Excellence (CfE). Although it offers translations and is ultimately aimed at higher income countries, there may be difficulties accessing it in low-income countries and those in digital poverty, especially when internet access is limited or there is poor internet connection.

The AMR learning resource has reliable, easily accessible content, which is freely downloadable to anyone interested in teaching from the e-Bug website. It is interactive and engaging, encouraging curiosity. The content and complexity of information presented in this resource are accurate to the target audience's level. This resource educates children in AMR but may provide messages that students can take home to help educate family and carers.

Although the resource is available to be used freely, awareness of its presence is essential if it is to be used in practice. This was the gap noted by the AMT in this area, and without AMR education being mandated in schools, the use of it relies on voluntary time and programmes, such as from university students. Therefore, consistency over geographical location will vary. This can be demonstrated even locally, where initially Aberdeen city schools were selected, due to ease of volunteer travel. Remote and rural schools are less likely to be able to access outreach, particularly in areas such as northern Scotland where this pilot took place.

Although the base materials are free, some of the extension tasks would incur some cost, such as the ‘Growth of a bacterial lawn’ task that includes colonies of bacteria grown on agar plates before the lesson.

Overall, this resource is an excellent way to support community and public health initiatives in further educating about AMR, especially in areas with high antibiotic misuse. When delivered as part of a schools outreach programme, volunteers enjoyed the sessions and found them useful and rewarding (Table 1). School teachers found the lessons informative and enjoyable for the pupils, and volunteers would be welcomed again (Table 1). The materials supplemented current science lessons, which did not include AMR in the curriculum. At present, the use of e-Bug in Scotland is reliant on voluntary schemes and an awareness of the existence of e-Bug, along with a motivation to provide such education. There was a high administrative burden assigning schools and visits, which would need to be factored into any replicative programmes. Teachers also fed back that although the toy microbes used were very exciting, they were distracting, and as such, when running future training programmes, education specialists will be sought. The University of Aberdeen is currently working towards mandating this education in a Student Selected Component (SSC) for medical students. The limitation with this is that only medical students are targeted, rather than more broadly scientific, nursing and pharmacy students, who could all benefit from this opportunity.

**Table 1.** Feedback from university students and teachers

University students	Teachers
‘I thoroughly enjoyed delivering the lesson to the two classes.’	‘We would be really happy to support more students coming in to do similar projects in the future with our learners. Only improvement (if this could be classed as one) would be for them to come in for more than one day, even for part of a day, so they can engage with more learners!’
‘We had a great time at the school and thought things went very well! The children were so receptive to the teaching and engaged well with all of the activities. We also had a great question time at the end. It was a pleasure for us to do this so thank you very much for giving us the opportunity!’	‘The activities were engaging and the pupils loved asking lots of questions. We are always telling them to wash their hands so it was very helpful for reinforcing that message! Thank you for organizing this for us and coming to visit. You are welcome back any time.’
‘The pupils were engaged and interested and when evaluating how much the pupils had learned, they were able say that they learnt something new.’	‘Many pupils found this quite interesting and it was pitched at the right level. There were plushy viruses and bacteria that pupils enjoyed looking at and which helped them visualize what was being talked about.’

We suggest that there are also wider benefits to the school pupils in terms of general science engagement and enthusiasm, career insights (getting to meet students training to be doctors) and also to the volunteers, in terms of developing skills in communication with children, medical education and teaching, and of communicating complex scientific ideas to the general public.

This pilot project should not be unique, given its mutual benefit to both university students and school-aged pupils.
